# Experimental and Kinetic Study on Lignin Depolymerization in Water/Formic Acid System

**DOI:** 10.3390/ijms18102082

**Published:** 2017-10-01

**Authors:** Qi Wang, Sipian Guan, Dekui Shen

**Affiliations:** 1 College of Metrology and Measurement Engineering, China Jiliang University, Hangzhou 310096, China; wangqi@cjlu.edu.cn; 2 Jiangsu Frontier Electric Power Technology Co., Ltd., Nanjing 211102, China; parallel79@163.com; 3 School of Energy and Environment, Southeast University, Nanjing 210096, China

**Keywords:** lignin, solvolysis, microwave heating, aromatic compounds, kinetic model

## Abstract

Microwave-assisted depolymerization of black-liquor lignin in formic acid was studied, concentrating on the yield of liquid fractions as bio-oil 1 (mainly aromatic monomers) and bio-oil 2 (mainly aromatic oligomers) and the distribution of the specific compositions. Bio-oil 1 (9.69%) and bio-oil 2 (54.39%) achieved their maximum yields under 160 °C with the reaction time of 30 min. The chemical compositions of bio-oil 1 and bio-oil 2 were respectively identified by means of Gas Chromatography-Mass Spectrometer (GC-MS) and Matrix-assisted laser desorption/ionization time-of-flight mass spectrometry (MALDI-TOF MS). Ethanone, 1-(4-hydroxy-3-methoxyphenyl) and Ethanone, 1-(4-hydrox-3,5-dimethoxyphenyl) were evidenced to be the two prominent compounds in bio-oil 1. Production of aromatic oligomers with the molecular weight of 328, 342, 358, 378, 394, 424 and 454 identified by MALDI-TOF MS was substantially tuned with the reaction temperature. A two-separate-stage kinetic model was proposed to describe the acidic solvolysis of lignin assisted by microwave heating, where the first stage is dominated by the depolyerization of lignin to monomers and oligomers with the activation energy of 40.27 kJ·mol^−1^, and the second stage with the activation energy of 49.18 kJ·mol^−1^ is mainly ascribed to the repolymerization of first-stage produced compounds.

## 1. Introduction

Lignin, rich in the natural plant cell-wall (15–35 wt %), has a three-dimensional (3D) chemical structure polymerized by three kinds of phenyl propane units through ether bonds or carbon-carbon linkages [1,2,3]. Utilization of lignin for producing value-added renewable chemicals attracts more and more attentions due to its outstanding characteristics as being renewable, low-cost, existing in large amount annually (such as residue from pulping industry and hydrolysis of biomass), and having an abundant content of phenyl units [4,5,6,7].

Solvolysis (thermal degradation of solid in the specific solvent) is one of the applicable methods for producing value-added chemicals. Aromatic compounds including catechol, phenol and methyl phenol were observed as the primary liquid products from the degradation of alkali lignin in supercritical water [8]. Similar products (such as guaiacol, vanillin and vanillic acid) were identified by Pińkowsk et al., finding that both depolymerization of lignin and repolymerization of the produced fragments were enhanced with the increased temperature [9]. The amount of four monophenols (4-ethylphenol, guaiacol, 4-vinylphenol, 4-vinylguaiacol, and 4-ethylguaiacol) was up to 30% of all identified compounds from degradation organosolv lignin in ethanol [10], stating that the lignin depolymerization were subjected to the cleavage of aryl–O ether bonds and decarbonylation reaction. It needs to be noted that most of the previous studies on solvolysis of lignin is concentrated on the effect of solvent, reaction time, temperature, and the addition of catalyst species, but rarely on that of heating method. Thermal conversion of biomass assisted by microwave heating attracts specific attentions due to its advantages, such as a fast heating rate and high heating efficiency over the traditional electrical heating [11,12,13]. The chemical bonds in macromolecules of biomass can be easily volatile and fractured in the microwave field, leading to the relatively low requirement of experimental conditions for the degradation of lignin (such as low reaction temperature and short time). The maximum yield of liquid product from the degradation of lignin in formic acid under microwave heating was achieved at 150 °C within 30 min, which is considered to be more moderate than that under electrical heating (280 °C and 2 h) [14]. Rahimi et al. [15] reported that the depolymerization of the oxidized lignin in formic acid could produce more than 60 wt % yield of low-molecular-mass aromatics. The depolymerization process proceeded sequentially via formylation, elimination, and hydrolysis. Formic acid did not act as a source of hydrogen-donor in the transfer reactions, but promoted the elimination reaction through the anaylysis of density functional theory (DFT) study [16]. The mechanism on solvolysis of lignin under microwave heating is still ambiguous, limiting its application for efficient production of value-added chemicals.

In order to fill the knowledge gap, depolymerization of lignin in formic acid assisted by microwave heating was investigated, concerning the yield of liquid products and distribution of aromatic monomers and oligomers. The possible mechanism of lignin degradation against temperature and time will be discussed regarding the formation of specific phenolic compounds. A two-separate-stage kinetic model is proposed for describing lignin depolymerization and secondary repolymerization in formic acid under microwave heating.

## 2. Results and Discussion

### 2.1. Yield of Liquid Products

**Figure 1:**
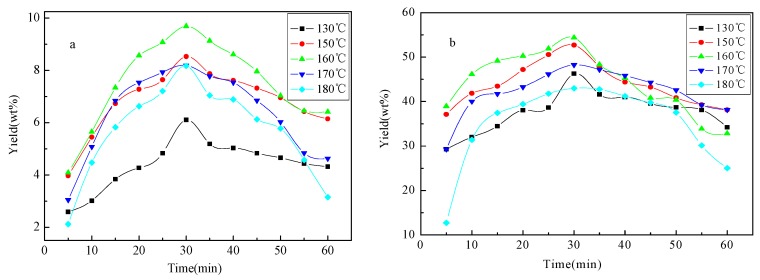
Yield of liquid products under different reaction temperatures and reaction times. (**a**) yield of bio-oil 1; (**b**) yield of bio-oil 2.

Yield of bio-oil 1 and bio-oil 2 against reaction time (5–60 min) under different temperatures (130, 150, 160, 170 and 180 °C) is shown in Figure 1. The yield of bio-oil 1 and bio-oil 2 is initially increased with the reaction time, achieved the maximum value around 30 min, and then decreased under all experimental temperatures. A similar trend is also observed for that against temperature, achieving the maximum yield at 160 °C for both bio-oil 1 (9.69%) and bio-oil 2 (54.39%). Much higher yield of low-molecular-mass aromatics (61.2% of the original aspen lignin) was achieved by Rahimi et al. [15] where the oxidized lignin was subjected to the formic acid/sodium formate reaction conditions at 110 °C for 24 h. The dominance of primary cracking of lignin to bio-oil 1 and bio-oil 2 over repolymerization of fragments in liquid phase is substantially confined by the reaction time and temperature under microwave heating.

### 2.2. Distribution of Aromatic Monomers in Bio-Oil 1

**Figure 2:**
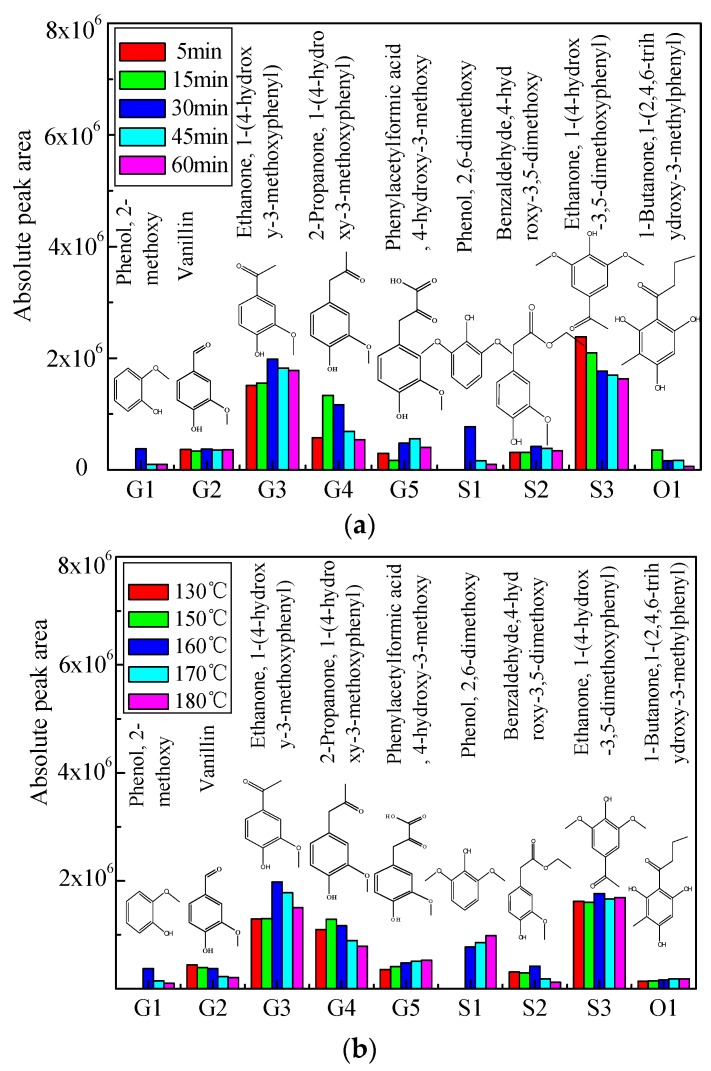
(**a**) Distribution of aromatic monomers in bio-oil 1 for different reaction times under 160 °C; (**b**) Distribution of aromatic monomers in bio-oil 1 for different reaction temperatures at 30 min.

The prominent aromatic monomers in *bio-oil 1* from acidic solvolysis of lignin under different reaction times and reaction temperatures were identified by GC/MS, the distribution of which is shown in Figure 2a,b in terms of absolute peak area. The yield of vanillin (G2) and Benzaldehyde, 4-hydroxy-3,5-dimethoxy (S2) were not notably influenced by reaction time (Figure 2a). Ethanone, 1-(4-hydroxy-3-methoxyphenyl) (G3), Phenol, 2-dimethoxy (G1), and 2,6-dimethoxy (S1) achieved their maximum yield at around 30 min, while 2-propanone, 1-(4-hydroxy-3-methoxyphenyl) (G4) reached its maximum production at 15 min. Yield of Ethanone, 1-(4-hydrox-3,5-dimethoxyphenyl) (S3) and 1-Butanone, 1-(2,4,6-trihydroxy-3-methylphenyl) (O1) was decreased with the reaction time under 160 °C, probably due to the intensive repolymerization reaction under longer residence time.

Comparatively, the yield of Ethanone, 1-(4-hydrox-3,5-dimethoxyphenyl) (S3), and 1-Butanone, 1-(2,4,6-trihydroxy-3-methylphenyl) (O1) is not notably influenced by reaction temperature at 30 min (Figure 2b). Phenol, 2-dimethoxy (G1) and ethanone, 1-(4-hydroxy-3-methoxyphenyl) (G3) and benzaldehyde, 4-hydroxy-3,5-dimethoxy (S2) achieved their maxima under 160 °C, while 2-propanone, 1-(4-hydroxy-3-methoxyphenyl) (G4) reached the highest production under 150 °C. The yield of phenylacetylformic acid, 4-hydroxy-3-methoxy (G5) was increased with the reaction temperature due to the enhancement of the reaction between the side-chain on the primary fragments and formic acid. Similar trend can be found for that of 2,6-dimethoxy (S1) probably attributed to the consumption of benzaldehyde, 4-hydroxy-3,5-dimethoxy (S2) under the higher temperatures through the elimination of side chain.

### 2.3. Characterization of Aromatic Oligomers in Bio-Oil 2

**Figure 3:**
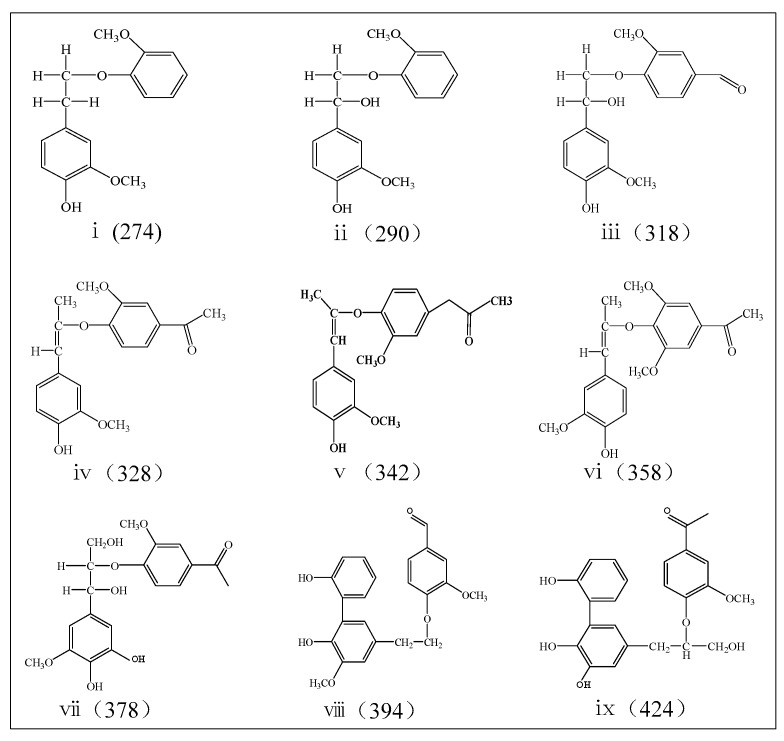
Proposed chemical structure of the aromatic oligomers detected by MALDI-TOF MS.

The outstanding peaks located in the range from 126 to 504 *m*/*z* of MALDI-TOF MS spectrum (Figures S1 and S2) were designated to the fragments of specific aromatic oligomers. The aromatic oligomers with molecular weight of 274, 290, 318, 328, 342, 358, 378, 394, and 424 are determined to be the remarkable compounds in bio-oil 2, the distribution in different reaction time and different reaction temperature are respectively shown in Table 1 and Table 2, the possible chemical structures of which are given in Figure 3. It can be found that the production of the aromatic oligomers reached the maximum 72.3% at 30 min, but decreased when the reaction time was more than 30 min. For the reaction time of 30 min, the aromatic oligomers reached the minimum production at 160 °C.

According to the speculation of oligomer chemical structures, oligomers i (274 *m*/*z*) and ii (290 *m*/*z*) (Table 1) are possibly related to the formation of G1 through the function of *C_α_–OH* linking pattern as described by Rahimi et al. [15]. The production of oligomer i (274 *m*/*z*) is narrowly varied from 7.5% to 8.6%, indicating that the stability of the produced oligomer i (274 *m*/*z*) is not notably influenced by reaction time, resulting in no significant formation of G1. The variation trend of G1 formation is in accordance with that of oligomer ii (290 *m*/*z*), both of which were decreased when the reaction time was more than 30 min. The formation of oligomers i (274 *m*/*z*) was gradually decreased with the increased temperature, and oligomer ii (290 *m*/*z*) reached the maximum production at 150 °C.

Yield of oligomer iii (318 *m*/*z*) gradually increased with the reaction time. The production of oligomer viii (394 *m*/*z*) exhibited a reverse trend against that of oligomer iii (318 *m*/*z*) when the reaction time was less than 45 min. It reveals a competitive mechanism for the formation of viii (394 *m*/*z*) and iii (318 *m*/*z*). The production of G2 is not correspondingly changed with reaction time, possibly revealing that oligomer iii (318 *m*/*z*) and viii (394 *m*/*z*) were mainly produced from the primary cracking of lignin macromolecules, but not from repolymerization of the produced fragments/derivatives (such as G2). The formation of oligomer viii (394 *m*/*z*) was increased with the increased temperature, while formation of G2 showed a reverse trend. It can be deduced that viii (394 *m*/*z*) might be produced by the repolymerization reaction of G2, since formation of G2 was evidence to be dominated during formic-acid-catalyzed lignin depolymerization in presence of sodium formate [15].

The production of oligomer vii (378 *m*/*z*) and ix (424 *m*/*z*) was first decreased and then increased with the increased reaction time, achieving the minimum value of 1.8% in 45 min and 4.2% in 30 min, respectively. Variation of the production of ethanone, 1-(4-hydroxy-3-methoxyphenyl) in bio-oil 1 (G3) (Figure 1) is found to be in a reverse trend with that of the oligomer vii (378 *m*/*z*) and ix (424 *m*/*z*) (Table 1). The oligomer vii (378) produced from the primary cracking of lignin should be one of the important precursors for the formation of G3 due to the effect of C_α_-O transformation as described by Rahimi et al. [15]. Oligomer vii (378 *m*/*z*) and ix (424 *m*/*z*) show the revise trend with the increased reaction temperature, showing the competitive formation relationship between them. The production of oligomer iv (328 *m*/*z*) shows the same trend with that of ethanone, 1-(4-hydroxy-3-methoxyphenyl) in bio-oil 1 (G3), resulting in no significant contribution for the formation of G3.

The other two prominent oligomers—v (342 *m*/*z*) and vi (358 *m*/*z*)—are considered to be associated with the production of 2-propanone, 1-(4-hydroxy-3-methoxyphenyl) (G4) and ethanone, 1-(4-hydroxy-3,5-dimethoxyphenyl) (S3). No remarkable trend between the formation of S3 and oligomer vi (358 *m*/*z*) can be found, except that both achieved maximum production in 5 min. The production of oligomer v (342 *m*/*z*) was not changed with increased reaction time, implying that oligomer v (342 *m*/*z*) might be produced from the primary cracking of lignin macromolecules without significant contribution for the formation of G4. The formation of S3 was not remarkably changed with the increased reaction temperature, while oligomer vi (358 *m*/*z*) first decreased and then increased with the temperature. This hints that there might not be significant reaction relationship between oligomer vi (358 *m*/*z*) and S3.

### 2.4. Kinetics of Microwave-Assisted Solvolysis of Lignin

**Scheme 1:**
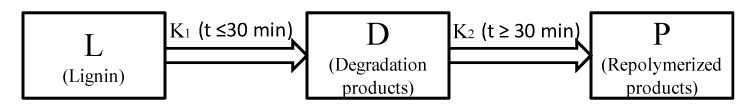
The two-separate-stage kinetic model of lignin depolymerization in formic acid assisted by microwave heating.

The depolymerization process of lignin in formic acid can be considered as a degradation step competing with the condensation step. Lignin was primarily degraded to small fragments (including monomers and oligomers), which can be further condensed into macromolecular substances (such as macromolecules and char). According to the yield variation of liquid products (bio-oil 1 and bio-oil 2) under different reaction times and temperatures in Figure 1a,b, reactions for lignin depolymerization in formic acid assisted by microwave heating can be divided into two separated stages, as shown in Scheme 1. The first stage (K_1_) is the lignin depolymerized to liquid products within the first 30 min, while the second stage (K_2_) is the the repolymerization of those produced small fragments to macromolecules after 30 min.

The reaction rate of the first stage reaction can be expressed as follow:

(1) $$\frac{d[L]}{dt}=-k_{1}\left[L\right]$$

where [*L*] is the mass fraction of lignin. The depolymerization was predominant during the deploymerization process of lignin. Taking into account that *r*_1_ *>> r*_2_ (where *r*_1_ and *r*_2_ are the formation rates for *D* and *P*, respectively) and (*−d*[*L*]*/dt* *≈ d*[*D*]*/dt*), Equation (1) can be rewritten as follows:

(2) $$\frac{d[D]}{dt}=k_{1}[L]$$

Since

(3) $$\left[L]=[L_{0}]-[D\right]$$

where [*L_0_*] is initial mass percent concentration of lignin.

(4) $$\left[L_{0}]\approx [D_{\infty }\right]$$

where [*D_∞_*] is the mass fraction of liquid products when the lignin is fully degraded into liquid products. Therefore, Equation (2) can be rewritten as follows:

(5) $$\frac{d[D]}{dt}=k_{1}([D_{\infty }]-[D])$$

The integration of Equation (5) allows the expression for linear dependence of [*D*] and the reaction time as follows:

(6) $$\ln([D_{\infty }]-[D])=\ln[D_{\infty }]-k_{1}t$$

Similarly, the reaction rate of the second stage reaction may be expressed as follows:

(7) $$\frac{d[P]}{dt}=-k_{2}[P]$$

where [*P*] is the mass fraction of the produced macromolecular substance. The repolymerization is dominated in the lignin depolymerization after 30 min. Taking into account that *r*_2_ *>> r*_1_ (where *r*_1_ and *r*_2_ are the formation rates of *D* and *P*, respectively) and (*−d*[*D*]*/dt* *≈ d*[*P*]*/dt*), Equation (7) can be rewritten as follows:

(8) $$\frac{d[D]}{dt}=-k_{2}[P]$$

Since

(9) $$\left[P]=[L_{0}]-[D\right]$$

where [*D_0_*] is [*D_l_*]. Equation (8) can be rewritten as follows:

(10) $$\frac{d[D]}{dt}=-k_{2}([D_{\infty }]-[D])$$

The integration of Equation (10) allows the expression for linear dependence of [*D*] and the reaction time as follows:

(11) $$\ln([D_{\infty }]-[D])=\ln[D_{\infty }]+k_{2}t$$

**Figure 4:**
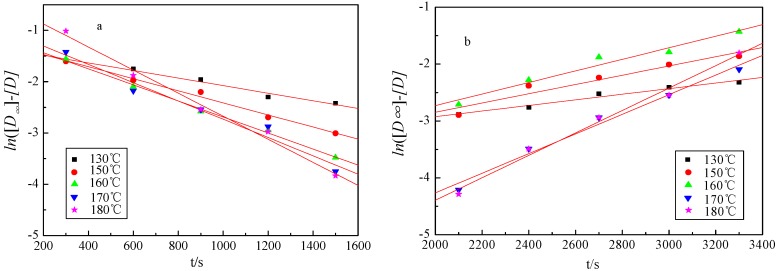
The linear fitting curve for the two-separate-stages kinetic model. (**a**) The first stage; (**b**) the second stage.

The yield variation of liquid products against reaction time and temperature is shown in Figure 2a. According to the Equations (6) and (11), the plot of ln([*D_∞_*] − [*D*]) against the reaction time could give the determination of the reaction constant [*k*] for *k*_1_ and *k*_2_ (Figure 4 and Table 3). It can be found that the curves presented good linear relationship, while the coefficient values are all greater than 0.98 (Table 3).

It was proved that the lignin degradation can follow the Arrhenius Equation [17]. Therefore, the kinetic parameters of the two-stage kinetic model for lignin depolymerization in formic acid can be achieved through the Arrhenius Equation as follows:

(12) $$\ln k=-\frac{E_{a}}{RT}+\ln A$$

The activation energy (*E_a_*) and other kinetic parameters are given in Table 4. The activation energy (*E_a_*) of the first stage reaction is 33.67 kJ·mol^−1^, which is less than the second stage reaction (44 kJ·mol^−1^). The activation energy of lignin depolymerization was significantly changed with the lignin sources. It was reported that the activation energy of softwood lignin depolymerization was 37 kJ·mol^−1^ by Zhang et al. [17]. The activation energy for the solvolysis of lignin isolated by sulfuric acid was 46 kJ·mol^−1^ [4], and the activation energy of lignin oxidative solvolysis assisted by microwave irradiation was 67 kJ·mol^−1^ [18].

## 3. Materials and Methods

### 3.1. Materials

The condensed black liquor was obtained from a pulping company (Yueyang, China). The extraction and purification of lignin, analysis of relevant physical and chemical properties, including Elemental Analysis, Fourier Transform Infrared Analysis and the analysis of distribution of the main inter-unit linkages by NMR (the frequencies of *β−O−4″*, *β−β″*, and *β−5″* bonds) were all described in authors’ previously published work [19].

### 3.2. Methods

Microwave digestion instrument (MDS-6) was purchased from Xinyi Microwave Chemical Technology Co., Ltd. (Shanghai, China). Reaction pressure of the digestion tank (SG–70: 70 mL) should be operated under 4 MPa, while the temperature should be no more than 250 °C.

**Figure 5:**
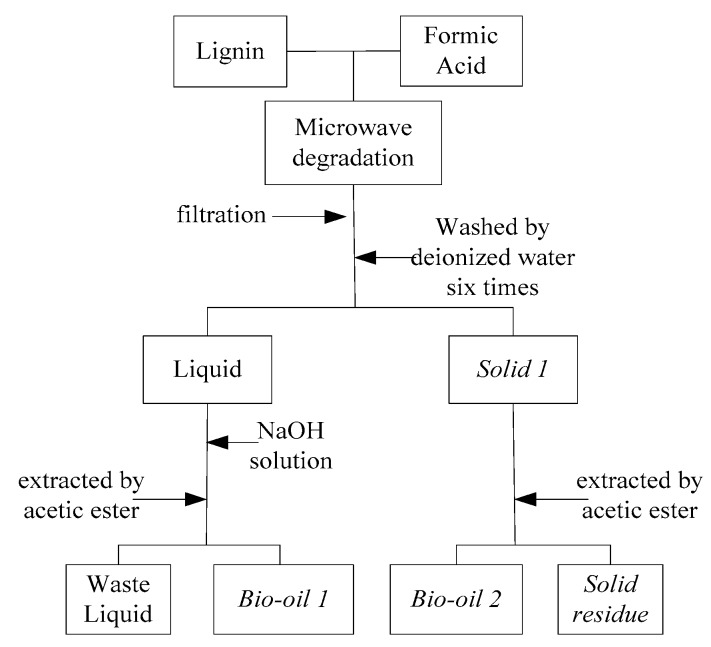
Schematics for the experimental process.

The experiment process was illustrated in Figure 5 and experimental runs were listed in Table 1 (reaction temperature: 110–180 °C and reaction time: 5–90 min). A 1 g lignin sample (150–200 mesh) was first mixed with 12 mL formic acid in the digestion tank. The microwave reactor was set in the fixed mode (power: 600 W, reaction temperature and reaction time), and then the digestion tank was placed in microwave reactor and started to be heated. After reaction, the digestion tank was cooled to room temperature by air. The liquid-solid mixture in the tank was filtered for separating the liquid product and solid residue (as solid 1 in Figure 1). Raw liquid product was reacted with a suitable amount of NaOH solution until the pH value was around 6. The liquid was then extracted by ethyl acetate, achieving bio-oil 1 which is mainly composed of aromatic monomers (phenolic compounds). Raw solid residue (solid 1) was washed/extracted by ethyl acetate, achieving solid residual and bio-oil 2. The yields of bio-oil 1 and bio-oil 2 were calculated gravimetrically referring to the initial lignin weight.

The bio-oil 1 mainly composed of phenolic monomeric compounds was identified by GC-MS (Agilent 7890A-5975C, Los Angeles, CA, USA). The operating conditions were as follows: the injector temperature was 300 °C; TR-35MS capillary column was used as the chromatographic separation; the oven temperature was programmed from 40 °C (kept for 3 min) to 180 °C (kept for 2 min) with a 5 °C/min heating rate and then up to 280 °C (kept for 2 min) with a 10 °C/min heating rate; and the mass spectra were operated in electron ionization mode at 70 eV and obtained from *m*/*z* 50 to 850. The chromatographic peaks were identified according to the National Institute of Standards and Technology (NIST) MS library, as well as previously published works [2,20,21].

The molecular weight distribution of oligomers in bio-oil 2 was determined by MALDI-TOF MS. The irradiation source of MALDI-TOF MS (AB SCIEX MALDI TOF∕TOFTM5800 Analyzer, New York, USA) was ND: YAG laser with a wavelength of 335 nm and the length of one laser pulse was 3 ns. The measurements were carried out as follows: polarity-negative, flight path-linear, mass-high (20 kV acceleration voltage), and 50–150 pulses per spectrum. CHCA (α-Cyano-4-hydroxycinnamic acid) was used as matrix media in the analysis. The molecular weight of aromatic oligomers can be obtained from the analysis of MALDI-TOF MS spectrum, together with their distribution in bio-oil 2 determined by the relative peak area. Chemical structure of the aromatic oligomers was speculated according to the specified molecular weight, in order to gain the formation information of aromatic monomers identified in bio-oil 1 and their kinetic relationship with oligomers.

## 4. Conclusions

The yield of bio-oil 1 and bio-oil 2 from lignin depolymerization in formic acid assisted by microwave heating reached the maximum value of 9.69% and 54.39%, respectively, within 30 min at 160 °C. Aromatic oligomers with molecular weights of 328 *m*/*z*, 342 *m*/*z*, 358 *m*/*z*, 378 *m*/*z*, 394 *m*/*z*, 424 *m*/*z*, and 454 *m*/*z* were characterized by MALDI-TOF MS. The production of the most detected oligomers was first decreased with the reaction temperature and then increased. A two-separate-stage kinetic model was employed to describe the Microwave-assisted acidolysis of lignin, achieving that the activation energy for the first stage as lignin depolymerization to fragments was 33.67 kJ·mol^−1^, and that for the second stage as the dominant repolymerization of the fragments was 43.87 kJ·mol^−1^.

## Figures and Tables

**Table 1:** Distribution of the aromatic oligomers identified by Matrix-assisted laser desorption/ionization time-of-flight mass spectrometry (MALDI-TOF MS) from lignin degradation at different reaction times under 160 °C.

No.	Molecular Weight ^a^	Peak (*m*/*z*)	Relative Peak Area ^b^ (%)				
			5 min	15 min	30 min	45 min	60 min
i	274	272–274	8.0	7.5	8.6	8.1	8.0
ii	290	283–294	0	5.0	5.7	3.5	3.0
iii	318	311–318	4.5	7.3	8.4	8.6	9.1
iv	328	319–328	11.3	11.6	16.4	12.5	8.2
v	342	330–342	6.3	6.4	6.1	6.7	6.1
vi	358	350–358	13.9	8.5	9.5	8.6	9.7
vii	378	368–378	4.0	3.8	2.5	1.8	2.6
viii	394	384–394	14.5	11.5	10.9	6.5	7.0
ix	424	424	8.9	5.9	4.2	5.0	5.5
Total			71.4	67.5	72.3	61.3	59.2

^a^ the fragments identified by MALDI-TOF MS with the m/z around the main peak were considered to be designated to the same oligomer molecule, and the absolute peak area of the determined molecule can be calculated as the sum of that of these fragments. ^b^ according to the distribution of MALDI-TOF MS spectrum for bio-oil 2, the integration range for all fragment peaks was set to be 253–504 *m*/*z* in order to give the calculation of relative peak area of the specific oligomer.

**Table 2:** Distribution of the aromatic oligomers identified by MALDI-TOF MS from lignin degradation under different reaction temperatures with the reaction time of 30 min.

No.	Molecular Weight ^a^	Peak (*m*/*z*)	Relative Peak Area ^b^ (%)				
			130 °C	150 °C	160 °C	170 °C	180 °C
i	274	272–274	9.4	8.9	8.6	8.3	10.4
ii	290	283–294	4.5	7.5	5.7	4.3	3.4
iii	318	311–318	7.9	7.9	8.4	7.0	6.4
iv	328	319–328	19.3	18.8	16.4	11.1	1.6
v	342	330–342	9.4	9.2	6.1	7.1	9.8
vi	358	350–358	11.5	6.8	9.5	10.5	14.8
vii	378	368–378	5.1	4.8	2.5	4.2	11.9
viii	394	384–394	5.9	6.0	10.9	13.8	19.4
ix	424	424	2.4	3.1	4.2	10.2	8.0
Total			75.4	73	72.3	76.5	85.7

^a^ the fragments identified by MALDI-TOF MS with the m/z around the main peak were considered to be designated to the same oligomer molecule, and the absolute peak area of the determined molecule can be calculated as the sum of that of these fragments. ^b^ according to the distribution of MALDI-TOF MS spectrum for bio-oil 2, the integration range for all fragment peaks was set to be 253–504 *m*/*z* to give the calculation of relative peak area of the specific oligomer.

**Table 3:** The calculated constants and correlation coefficient under different reaction temperatures.

Temperature (°C)	The First Stage		The Second Stage	
	*k* (L·mol^−1^·s^−1^)	*R*	*k* (L·mol^−1^·s^−1^)	*R*
130	0.000739	0.99036	0.000493	0.98531
150	0.00118	0.99535	0.000814	0.9655
160	0.00156	0.99823	0.00102	0.98169
170	0.00179	0.98472	0.00173	0.99257
180	0.00225	0.99453	0.00197	0.99391

**Table 4:** The parameters for the two-separate-stage kinetic model.

Reaction	The Linear Fitting Equation	*R*	*E_a_* (kJ·mol^−1^)	*A* (L·mol^−1^·s^−1^)
The first stage	ln*K =* 2.8430 − 4049.35*/T*	0.99792	33.67	17.1672
The second stage	ln*K =* 5.4203 − 5276.81*/T*	0.98374	43.87	225.9469

## Supplementary Materials

Supplementary materials can be found at www.mdpi.com/1422-0067/18/10/2082/s1.
