# Supplementary material for: Experimental and Kinetic Study on Lignin Depolymerization in Water/Formic Acid System
Source: Int J Mol Sci. 2017 Oct 1;18(10):2082. doi: 10.3390/ijms18102082 (PMC5666764; doi:10.3390/ijms18102082)
Supplement: Supplementary file 1 [file ijms-18-02082-s001.pdf]

Experimental and Kinetic Study on Lignin Depolymerization in Water/Formic Acid System

Qi Wang [1], Sipian Guan [2], and Dekui Shen [3,*]

[1] College of Metrology and Measurement Engineering, China JiLiang University, Hangzhou 310096, China; wangqi@cjlu.edu.cn
[2] Jiangsu Frontier Electric Power Technology Co., Ltd., Nanjing 211102, China; parallel79@163.com
[3] School of Energy and Environment, Southeast University, Nanjing 210096, China
* Correspondence: 101011398@seu.edu.cn; Tel.: +86-025-8379-4744

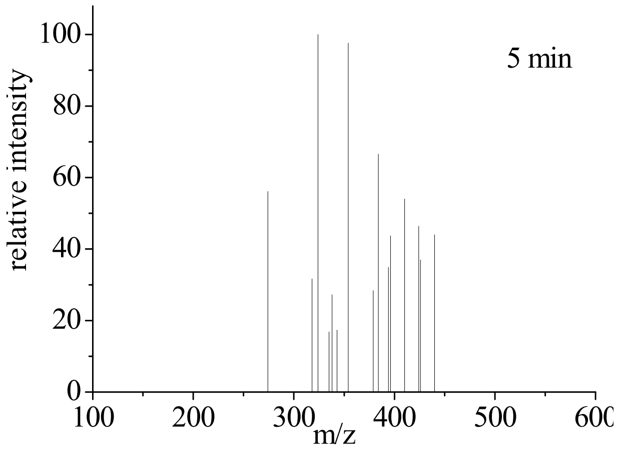

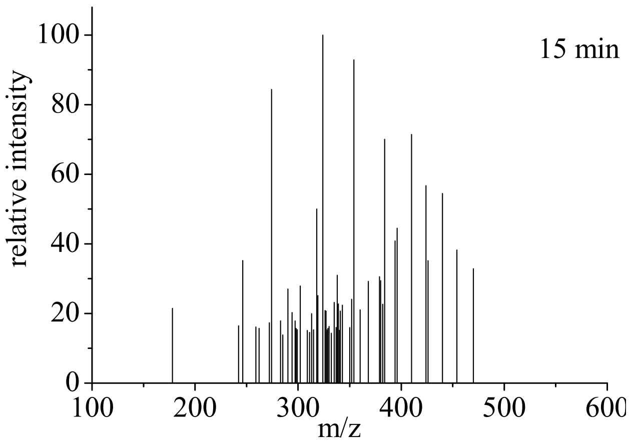

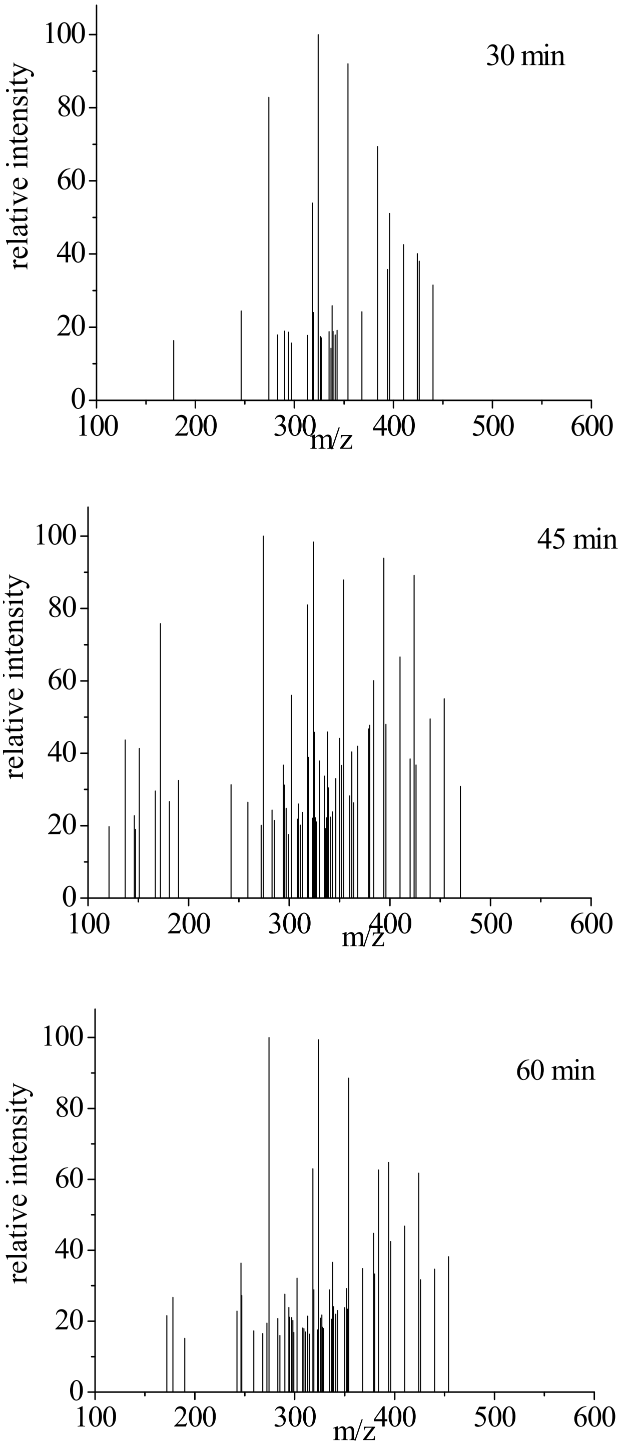

Fig. S1: MALDI-TOF MS spectra of *bio-oil 2* under different reaction times at 160 ^{o}C

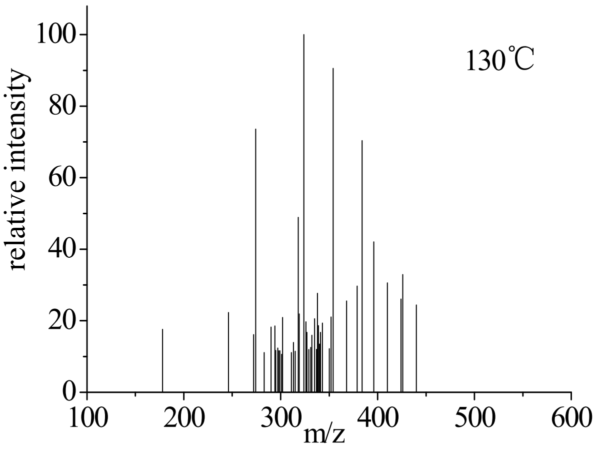
130℃
relative intensity
m/z
0
20
40
60
80
100
100
200
300
400
500
600

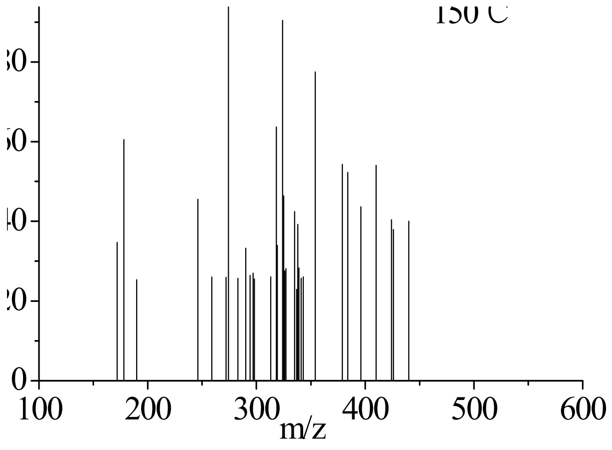
m/z
0
100
200
300
400
500
600

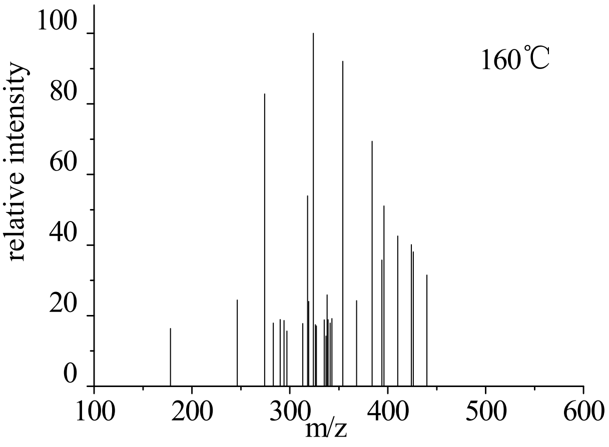
160℃
relative intensity
m/z
0
20
40
60
80
100
100
200
300
400
500
600

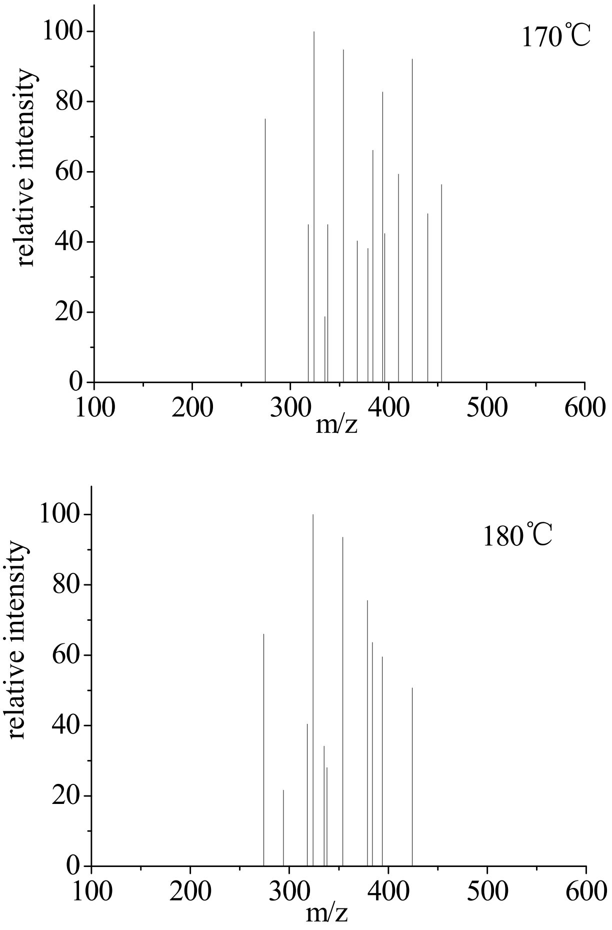

Fig. S2: MALDI-TOF MS spectra of *bio-oil 2* at different temperature with the reaction time of 30 min
